# CTP Synthase 2 From *Arabidopsis thaliana* Is Required for Complete Embryo Development

**DOI:** 10.3389/fpls.2021.652434

**Published:** 2021-04-15

**Authors:** Daniel Hickl, David Scheuring, Torsten Möhlmann

**Affiliations:** ^1^Plant Physiology, University of Kaiserslautern, Kaiserslautern, Germany; ^2^Plant Pathology, University of Kaiserslautern, Kaiserslautern, Germany

**Keywords:** *Arabidopsis*, nucleotides, *de novo* synthesis, CTP synthase, embryo development

## Abstract

Pyrimidine *de novo* synthesis is an essential pathway in all organisms. The final and rate-limiting step in the synthesis of the nucleotide cytidine triphosphate (CTP) is catalyzed by CTP synthase (CTPS), and *Arabidopsis* harbors five isoforms. Single mutant lines defective in each one of the four isoforms do not show apparent phenotypical alterations in comparison to wild-type plants. However, *Arabidopsis* lines that contain T-DNA insertions in the *CTPS2* gene were unable to produce homozygous offspring. Here, we show that *CTPS2* exhibits a distinct expression pattern throughout embryo development, and loss-of-function mutants are embryo lethal, as siliques from *+/ctps2* plants contained nearly 25% aborted seeds. This phenotype was rescued by complementation with *CTPS2* under control of its endogenous promoter. CTPS2::GFP lines revealed expression only in the tip of columella cells in embryo root tips of the heart and later stages. Furthermore, *CTPS2* expression in mature roots, most pronounced in the columella cells, shoots, and vasculature tissue of young seedlings, was observed. Filial generations of *+/ctps2* plants did not germinate properly, even under external cytidine supply. During embryo development, the *CTPS2* expression pattern resembled the established auxin reporter DR5::GFP. Indeed, the cloned promoter region we used in this study possesses a repeat of an auxin response element, and auxin supply increased *CTPS2* expression in a cell-type-specific manner. Thus, we conclude that CTPS2 is essential for CTP supply in developing embryos, and loss-of-function mutants in *CTPS2* are embryo lethal.

## Introduction

Nucleotides are essential building blocks for the production of nucleic acids. In addition, nucleotides represent the main energy carriers in biochemical reactions and function as nitrogen, carbon, or phosphate source under nitrogen-limiting conditions and as cofactors in phospholipid biosynthesis. Due to their chemical structure, nucleotides are divided into purines and pyrimidines (Kinney, 1993; Moffatt and Ashihara, 2002; Zrenner et al., 2006). In plants, nucleotide metabolism consists of (i) *de novo* synthesis, (ii) salvage, and (iii) degradation (Zrenner et al., 2006). Pyrimidine *de novo* synthesis consists of six enzymatic steps distributed to the chloroplast, cytosol, and mitochondria, ending up with the production of uridine monophosphate (UMP) in the cytosol. This intermediate is phosphorylated by UMP kinase to UDP. Uridine mono-, di-, and triphosphates are equilibrated by nucleoside diphosphate kinases. The last step of pyrimidine *de novo* synthesis is the amination of UTP to cytidine triphosphate (CTP), conducted exclusively by CTP synthases (CTPS) (Moffatt and Ashihara, 2002; Zrenner et al., 2006; Witz et al., 2012). CTP synthases represent a conserved enzyme family found across kingdoms. The demand of CTP as part of DNA is especially high during cell division and in developing tissues. Therefore, CTPS activity was described to be regulated on different levels, e.g., posttranslationally (by phosphorylation) and allosterically (via GTP), or feedback inhibited by its product CTP (Levitzki and Koshland, 1972; Chang and Carman, 2008). A further enzymatic regulation is the polymerization to filaments, which was studied in several organisms like *Escherichia coli*, *Saccharomyces cerevisiae*, *Drosophila melanogaster*, and *Homo sapiens* (Liu, 2010; Barry et al., 2014; Noree et al., 2014; Lynch et al., 2017). Plant CTPS were first described in *Arabidopsis thaliana* (hereafter referred to as *Arabidopsis*) by Daumann et al. (2018), describing features of five isoforms including the ability of filament formation. The isoforms show tissue-specific expression patterns, which are dynamic for CTPS1 and 4 under abiotic stresses (Hruz et al., 2008; Daumann et al., 2018). However, single knockout mutants did not show any phenotype under long- or short-day growth regimes, except for CTPS2. We were not able to produce homozygous T-DNA insertion lines for this isoform, indicating a special function during embryo development or germination (Daumann et al., 2018).

The six core enzymatic steps of pyrimidine *de novo* synthesis are facilitated by five enzymes, each encoded by a single gene. Homozygous knockout lines cannot be generated, indicating the essential nature of this pathway (Schröder et al., 2005). Antisense lines of aspartate transcarbamoylase (ATC) and dihydroorotase (DHO), which facilitate the second and third step in pyrimidine *de novo* synthesis, caused growth restrictions in *Solanum tuberosum*. Nevertheless, antisense lines with 20% residual ATC or DHO protein were viable and able to produce tubers. Furthermore, no changes in nucleotide pools were observed in fully developed tissues unless expression dropped below a threshold, pointing to an efficient nucleotide salvage in older plants (Schröder et al., 2005). A detailed analysis of the offspring of heterozygous loss-of-function mutants in enzymatic steps in purine and pyrimidine *de novo* synthesis and the plastidic pentose phosphate pathway (providing substrates for nucleotide *de novo* synthesis and salvage) revealed an arrest at early stages of embryo development (Andriotis and Smith, 2019). This observation underlines the importance of nucleotide homeostasis in those tissues. In line with these findings, a large set of genes encoding proteins in central pathways has been classified as essential for embryo development (*EMB* genes) including genes involved in nucleic acid synthesis (Meinke, 1985, 2020). Due to the fact that pyrimidine *de novo* synthesis is facilitated by enzymes, which are mainly encoded by single genes, it is not surprising that homozygous knockouts of any of these genes are not viable. Since *Arabidopsis* harbors five CTP synthase isoforms that theoretically could be redundant in function, it was surprising that no homozygous knockout for isoform two could be generated (Daumann et al., 2018).

Here, we report that CTPS2 is indispensable for proper embryo development, presumably because CTP synthesis from UTP is required in embryo tissues. Tissue-specific expression of *CTPS2* in embryos and seed abortion in +/*ctps2* siliques was observed in addition. Young seedling roots show a cell-type-specific expression as well, which is responsive to exogenously supplied auxin.

## Materials and Methods

### Plants and Growth Conditions

*Arabidopsis thaliana* ecotype Columbia [denominated as wild type (WT)] was used in this study together with *CTPS2* (At3g12670) T-DNA insertion lines from the GABI-Kat collection (Kleinboelting et al., 2012). These were GABI_032C02 and GABI_156G07, designated as *+/ctps2-1* and *+/ctps2-2*, respectively. Seeds of soil-grown plants were sown on standardized ED73 soil (Einheitserde und Humuswerke Patzer, Buchenberg, Germany), incubated for 48 h at 4°C for stratification and transferred to growth chambers under long day regime (14 h light/10 h dark). Growth conditions were light intensity of 120 μmol quanta m^–2^ s^–1^, temperature of 22°C, and humidity of 60%. Segregation and promoter analysis were conducted with surface-sterilized seeds on 1/2 Murashige–Skoog (MS) agar plates (Murashige and Skoog, 1962), supplemented with vitamins, 1% sucrose (w/v), 0.05% MES–KOH (w/v), pH 5.7, and 0.8% agar. Seeds were surface sterilized by the addition of 500 μl 70% ethanol supplemented with 0.1% Triton X-100 for 5 min on an end-over-end shaker. After 1 min centrifugation at 5,000 *g* for 1 min, supernatant was discarded, and seeds were washed twice with 100% ethanol for 1 min. The seed/ethanol solution was immediately pipetted on a sterile filter paper and dried in the airflow of a sterile bench.

### Construction of CTPS2::GFP/GUS and CTPS2 Complementation Lines

For the construction of CTPS2::GFP/GUS lines, 1,002 bp upstream of the start codon was PCR amplified using CTPS2_Promoter sense and antisense primers (Supplementary Table 1). Subsequently, the amplified sequence was separated on an agarose gel (1% w/v) and isolated by gel digestion with a NucleoSpin^®^ Extract II kit (Macherey Nagel, Düren). Afterward, att-sites were attached by another PCR reaction, the amplified fragments were isolated as described above and used for Gateway cloning (primers listed in Supplementary Table 1). Once integrated in the entry vector pDONR (Thermo Fisher Scientific, Waltham, MA, United States), another Gateway reaction inserted the construct into pBGWFS7.0 (Karimi et al., 2002). This construct allows to monitor ß-glucuronidase (GUS) activity and green fluorescent protein (GFP) fluorescence from the same construct and the same transformed lines. For simplicity, we either refer to CTPS2::GFP or CTPS2::GUS in corresponding experiments. Transformation into wild-type *Arabidopsis* plants was performed according to the floral inoculation procedure (Narusaka et al., 2012).

Complementation of *+/ctps2-1* plants used 1,864 bp upstream of the *CTPS2* start codon, i.e., the full upstream intergenic region plus the protein coding region including introns of *CTPS2* (Schwacke et al., 2003). The corresponding region was amplified by PCR with CTPS2_full-lenth sense and antisense primers (Supplementary Table 1) on wild-type genomic DNA (gDNA). Subsequently to PCR, the amplified sequence was inserted into the entry vector pDONR (Thermo Fisher Scientific, Waltham, MA, United States) as described above and further into pMDC123 for complementation of *+/ctps2* plants (Curtis and Grossniklaus, 2003). Error-free amplification and vector insertion were checked by enzyme digestion and Sanger sequencing (Seq-IT, Kaiserslautern Germany). Primers used for this are listed in Supplementary Table 1.

The *CTPS2* complementation construct was used for transformation of *+/ctps2-1* plants as described above.

### Light Microscopy and Sample Preparation

Siliques of soil-grown plants (WT and *+/ctps2-1*) were harvested 8–10 days after fertilization (DAF), longitudinal dissected and placed on a microscope slide, and covered with parafilm. Seed shape was investigated on 12–15 DAF siliques, which were dried for up to 7 days at room temperature. Siliques were carefully opened under the light microscope and placed on a microscope slide covered with parafilm for better grip. Herein, WT, *+/ctps2* plants, and *+/ctps2* plants complemented with endogenous CTPS2 were used. Images were taken with a Leica MZ10 F microscope equipped with a Leica DFC420 C camera.

### Confocal Laser Scanning Microscopy and Sample Preparation

Developing siliques of CTPS2::GFP promoter lines were harvested 1.5–8 DAF, dissected, seeds isolated, and transferred to 1.5-ml reaction tubes. After addition of 10 μl ddH_2_O per seed, embryos were carefully squeezed out with a potter. The embryo/water solution was transferred to microscope slides and immediately used for microscopy. For the investigation of roots of CTPS2::GFP plants, seeds were placed on 1/2 MS agar plates containing 1% agar and vertically positioned in the growth chamber. At the indicated time points, seedlings were mounted in propidium iodide (PI) solution (0.01 mg/ml) for cell wall staining, followed by confocal laser scanning microscopy. Images in Figures 3, 5, 6 were taken with a Leica TCS SP5II microscope. The excitation for GFP was 488 nm, and detection of emission was at 505–540 nm. Chlorophyll autofluorescence of embryos and PI fluorescence were detected at 514 nm emission and at 651–704 nm excitation through a HCX PL APO 63 × 1.2 W water immersion objective.

Images in Figure 4 and Supplementary Figures 1, 4 were acquired with a Zeiss LSM880, AxioObserver SP7confocal laser-scanning microscope (INST 248/254-1), equipped with a Zeiss C-Apochromat 40 × 1.2 W AutoCorr M27 water-immersion objective with fluorescence settings as given in Kaiser et al. (2019).

Images were processed using Leica software LAS X (version 3.3) or Zeiss software ZEN 2.3.

### Auxin Treatment

Surface-sterilized seeds of CTPS2::GFP reporter plants were placed on 1/2 MS agar plates and vertically grown for 7 days in a long-day chamber. Afterward, seedlings were transferred for 20 h to 1/2 MS agar plates containing 250 nM 1-naphthaleneacetic acid (NAA). NAA was solubilized in 100% dimethyl sulfoxide (DMSO) and added to lukewarm 1/2 MS media prior to pouring the plates. As control, 250 nM DMSO was added to another batch of plates, and the same number of seedlings was transferred to control conditions as for NAA treatment.

Fluorescence was detected with a Zeiss LSM880 as mentioned above. Images were processed using ImageJ (version 1.51j8), and the mean gray value is given from fluorescence of 10 trichoblast cells of five biological replicates.

### Statistical Analysis

All experiments were carried out at least three times. Box limits in the graphs represent 25th–75th percentile, the horizontal line the median, small light gray box the mean, and whiskers minimum to maximum values.

## Results

### Cytidine Supplemented Segregation Analysis of *+/ctps2* Seeds

**FIGURE 1:**
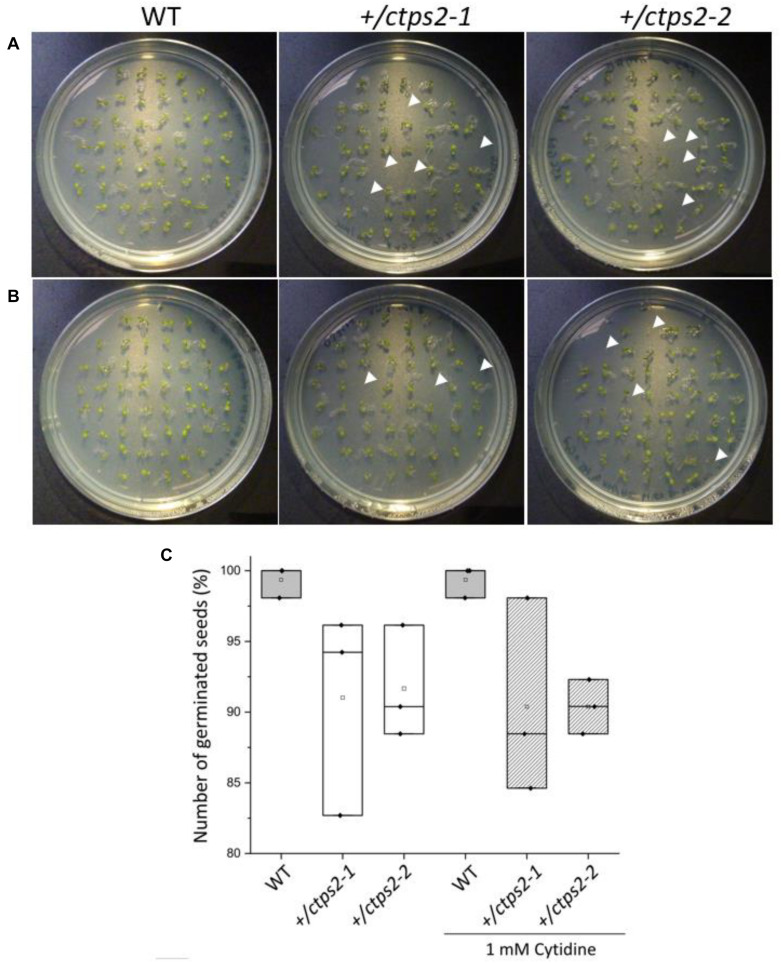
Germination of *+/ctps2* lines on 1/2 MS agar plates. Wild-type (WT) and *+/ctps2* seeds were placed on **(A)** 1/2 Murashige–Skoog (MS) agar plates or **(B)** on plates supplemented with 1 mM cytidine. White arrowheads indicate non-germinated seeds 5 days after transferring to growth chambers. **(C)** number of germinated seeds with and without application of 1 mM cytidine. A total of 52 seeds were plated for each genotype, and the experiment was repeated three times.

Previously, we identified two *CTPS2* T-DNA insertion lines from the Gabi-Kat collection, which were unable to produce homozygous offspring (Daumann et al., 2018). These heterozygous lines (GK_032C02 and GK_156G07) were designated as *+/ctps2-1* and *+/ctps2-2*, respectively. A former segregation analysis of these two lines showed a deviant germination pattern, compared to the expected 1:2:1 ratio for WT:heterozygous:homozygous offspring (Daumann et al., 2018). Therefore, we concluded that the crucial function of CTPS2 is probably in germination and/or embryo development. To confirm the before-mentioned irregular pattern, we repeated the experiment in our study and found that nearly 10% of *+/ctps2-1* and *+/ctps2-2* seeds, which were sown on 1/2 MS agar plates did not germinate compared to WT (Figures 1A,C), implicating a homozygous *CTPS2* knockout in these seeds. Since nucleotide *de novo* synthesis is very energy intensive, plants are able to recycle nucleosides via the salvage pathway (Zrenner et al., 2006). On this occasion, external supplied nucleosides can be imported by the equlibrative nucleoside transporter 3 (ENT3) for being phosphorylated to nucleotides (Traub et al., 2007). The supplementation of 1/2 MS agar plates with 1 mM cytidine for another segregation analysis results again in 10% non-germinated seeds of both *+/ctps2* lines, whereas WT seeds grew normally (Figures 1B,C). This finding suggests that the critical role of CTPS2 takes place in seed production rather than germination.

### Siliques of *+/ctps2* Plants Contain Nearly 25% Aborted Seeds

**FIGURE 2:**
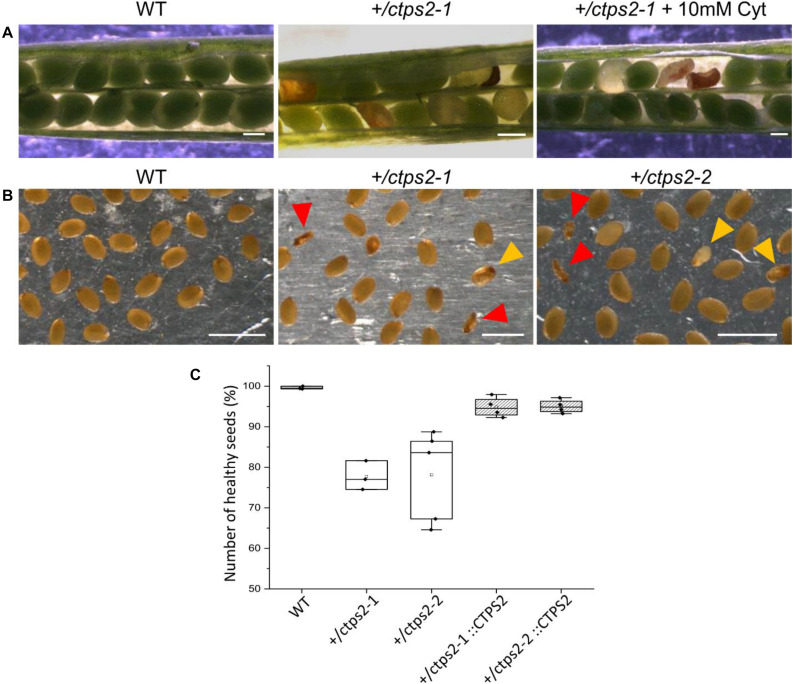
Identification of homozygous *ctps2* seeds. Siliques were opened 8–10 days after fertilization (DAF) from wild-type (WT) and *+/ctps2-1* plants. **(A)** irrigation of *+/ctps2-1* plants with 10 mM cytidine cannot rescue the aborted seed phenotype. Twelve DAF siliques were harvested and desiccated at room temperature, and seeds were collected using light microscopy. **(B)** red arrowheads point to collapsed seeds, while golden arrowheads indicate colorless seeds with nearly normal size, containing presumably embryos arrested early in development. Counting of seeds for WT, *+/ctps2*, and complemented *+/ctps2* plants. Scale bar = 0.25 mm in **(A)** and 1 mm in **(B)**. *n* = 3 for WT and *+ctps2-1* with a total of 38 and 40 siliques, respectively; *n* = 4 for *+/ctps2-1*::CTPS2 and *+/ctps2-2*::CTPS2 with a total of 38 and 36 siliques, respectively; *n* = 5 for *+/ctps2-2* with 52 siliques in **(C)**.

To investigate the seed development in detail, WT and *+/ctps2-1* seeds were sown on soil, and single plants were separated to individual pots. Heterozygous T-DNA insertion in *+/ctps2-1* was verified by PCR with gene-specific and T-DNA-specific primer combinations as given in Daumann et al. (2018). After the plants started reproductive growths, WT and *+/ctps2-1* siliques were harvested 8 days after fertilization (DAF). WT siliques contained healthy seeds with a deep green colored embryo inside, whereas *+/ctps2-1* siliques showed beneath healthy seeds, transparent, as well as collapsed brown colored seeds (Figure 2A). To check whether supplementation with pyrimidines can rescue the phenotype, the blossoms of three *+/ctps2-1* plants was removed except one inflorescence with closed buds. These plants were irrigated with water containing 10 mM cytidine until siliques attained 8 DAF. Nevertheless, embryo-free and collapsed seeds were still observable (Figure 2A), implicating that cytidine was not transported into the embryo or that the embryo is unable to recycle this compound. We assumed that the aborted seeds are homozygous for the T-DNA insertion and started to count seeds from *+/ctps2* plants. In both lines, seeds were found, which were completely collapsed or had a nearly normal size but were flat (Figure 2B). Both types were considered as aborted seeds. From a total of 2125 seeds in WT, 0.4% were aborted, whereas *+/ctps2-1* contained 22.3% and *+/ctps2-2* 21.9% aborted seeds in 2,142 and 2,475 seeds, respectively (Figure 2C). Thus, by counting aborted seeds in siliques from +/*ctps2* offspring, nearly the expected 25% of homozygous = non-viable seeds were observed. The reason why this is not reflected in our germination assay is because many aborted and collapsed seeds are lost or overlooked during harvest, seed cleaning, or sowing.

**FIGURE 3:**
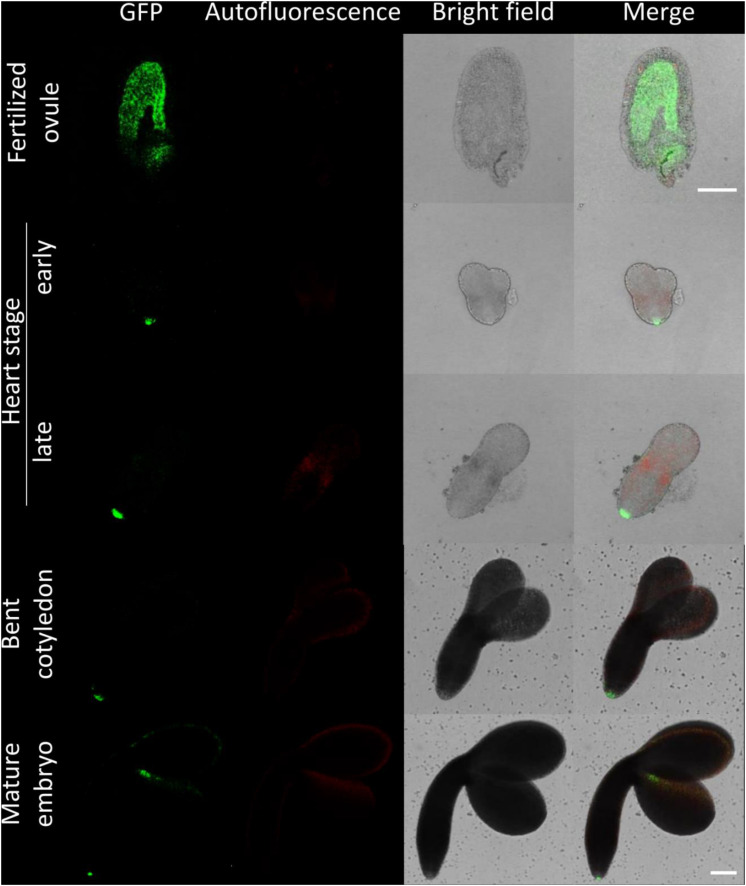
Fluorescence imaging of CTPS2::GFP lines in embryo cells. Siliques of soil-grown plants (lines CTPS2::GFP #10 and #15) were opened 2 days after fertilization (DAF) and between 6 and 8 DAF, and seeds were harvested. Ovules and embryos were isolated from seeds for confocal laser scanning microscopy. Typical images are shown. Scale bar = 100 μm.

**FIGURE 4:**
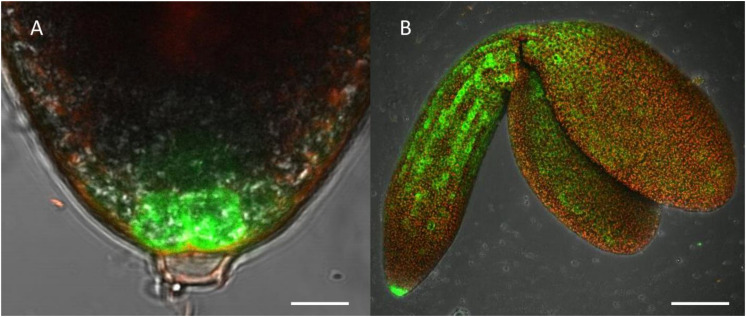
Highlight of CTPS2::GFP fluorescence imaging. Embryos were isolated from siliques 8 days after fertilization (DAF) for confocal laser scanning microscopy. For this, lines CTPS2::GFP #10 and #15 were used and typical images were selected. Scale bar = 10 μm in **(A)** and 100 μm in **(B)**. Maximum projection of 24 pictures in **(B)**.

**FIGURE 5:**
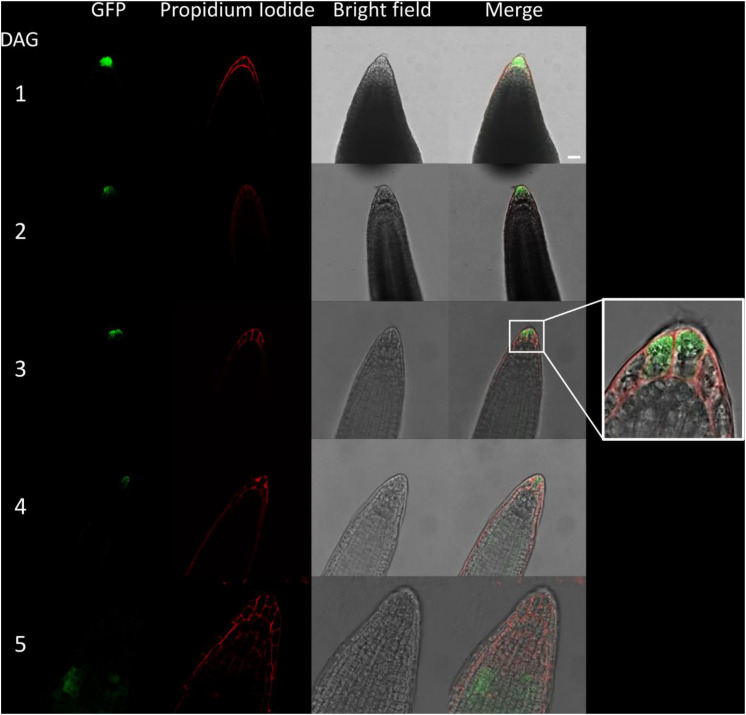
Fluorescence imaging of CTPS2::GFP lines in young seedling roots. Seedlings were grown on 1/2 Murashige–Skoog (MS) agar plates and at the indicated time points transferred to slides with propidium iodide containing water for cell wall staining and subsequent confocal laser scanning microscopy with a Leica TCS SP5II microscope. CTPS2::GFP lines #10 and #15 were used. Typical images are shown. Scale bar = 25 μm. DAG, days after germination.

**FIGURE 6:**
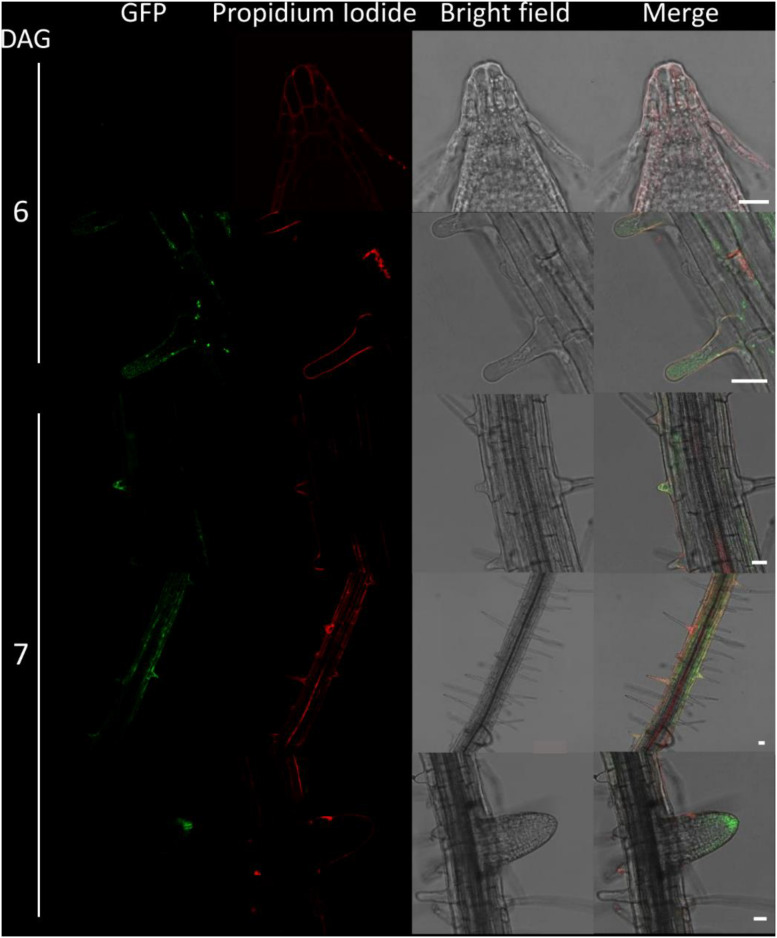
Fluorescence imaging of CTPS2::GFP lines in seedling roots. Growth and microscopy conditions as in Figure 5. Scale bar = 25 μm. DAG, days after germination.

Integrating the full protein coding sequence of *CTPS2* under control of the full endogenous promoter (1,864 bp upstream of ATG) into *+/ctps2* plants resulted in an average of only 5% aborted seeds in both lines demonstrating that the phenotype was at least partially complemented (Figure 2C). We also aimed to rescue the *+/ctps2* seed phenotype by expression of CTPS1-4 isoforms driven by the UBQ promoter as YFP-fusion constructs (already in our hands, described in Daumann et al., 2018). For this, +/*ctps2* plants were transformed, positive transformants screened for kanamycin resistance, and verified by PCR. Three plants per transformed construct and 4–10 siliques from the corresponding plants were inspected for WT-like seed development, but in no case complementation was successful. In addition, PCR inspection of transformed offspring of these transformants did not reveal homozygous T-DNA insertion in any case. Thus, CTPS2 driven by the endogenous promoter seems to be crucial for proper embryo development.

### CTPS2::GFP/GUS Lines Reveal Tissues-Specific *CTPS2* Expression During Embryo Development

For visualizing *CTPS2* promoter activity, 1,002 bp upstream of the start codon was used. This nucleotide sequence was cloned in pBGWFS7.0 (Karimi et al., 2002) and by this translationally fused to an eGFP and β-glucuronidase (GUS) tag. Five positive transformants were identified, and genomic integration of the construct was confirmed by PCR. A similar GUS staining pattern was observed in all five lines, and for further analysis, lines CTPS2::GFP/GUS #10 and #15 were selected.

Histochemical stainings of CTPS2::GUS lines revealed expression in roots, leaves, and flowers of *Arabidopsis* plants throughout development (Supplementary Figure 1). Five-day-old seedlings showed GUS staining in the root (especially in tips of primary and secondary roots), shoot apical meristem, leaf vasculature tissue, and filaments of flowers (Supplementary Figure 1).

Fertilized ovules at approximately 1.5 DAF showed strong CTPS2::GFP signals in the peripheral and chalazal endosperm but with strongest fluorescence signal in the peripheral endosperm (Figure 3). The *Arabidopsis* embryo develops to the heart stage 6 DAF, and from this, it develops quickly to the mature embryo until 8 DAF (Feng and Ma, 2017). Increased expression was observed from globular to heart stage embryos in a genome-wide expression survey (Winter et al., 2007). In this study, a maximal expression over all *Arabidopsis* tissues was observed at the curled cotyledon stage. We followed *CTPS2* expression during rapid development up to the mature embryo stage. In the early heart stage, we observed a punctate fluorescence pattern, preferentially in embryos columella cells. Even in lateral view, rotated 90°, the late heart stage embryo showed the same expression pattern, but the fluorescence signal was specifically localized to only two or four root tip cells (Figure 3). When the embryo develops to the bent cotyledon stage, the fluorescence signal remains strong in these columella cells. When the embryo reaches the mature stage, CTPS2::GFP fluorescence was observed also in other tissues than the columella additionally. Herein, promoter activity was observable in both cotyledons (Figure 3). Surprisingly, the strong fluorescence signal in embryo columella cells seems to occur only in two cells, where the former connection between embryo and suspensor cells has been (Figure 4A). Early embryo and also endosperm cells are connected to the maternal tissue via the suspensor to ensure nutrient and phytohormone supply by the mother plant (Bozhkov et al., 2005; Robert et al., 2018). Moreover, a maximum projection of the mature embryo revealed strong CTPS2::GFP fluorescence in root epidermis cells and also in the cotyledons (Figure 4B). Especially in root epidermis cells, it became obvious that the fluorescence signal was not distributed homogenously but seemed to be present in later atrichoblast or trichoblast cells (Figure 4B). These findings let us suggest that *CTPS2* is spatiotemporally expressed in columella cells of the developing embryo. After 7 DAF, the expression extends to further root and cotyledon tissues.

### CTPS2::GFP Fluorescence in Seedling Roots Is Restricted to Columella Cells and Trichoblasts

Due to the irregular pattern of *CTPS2* expression in the embryo, the roots of young, germinated seedlings were studied in more detail. One day after germination, the CTPS2::GFP fluorescence signal was very strong in root tip cells (Figure 5). In contrast to the images we took from developing embryos, roots were stained with the cell wall intercalating dye propidium iodide. Thereby, we found that CTPS2::GFP fluorescence was restricted to two central cells in the root tip (Figure 5). While the fluorescence signal intensity in central root tip cells decreased over time, it was completely absent from this cell type 5 days after germination (Figure 5). When the seedling becomes 5 days and older, the fluorescence signal shifts toward the elongation and later on toward the differentiation zone (Figures 5, 6). In the differentiation zone, *CTPS2* promoter activity was only found in young and developing root hair cells (trichoblasts) (Figure 6 and Supplementary Figure 2), thus pointing again to a strong spatiotemporal expression of *CTPS2* (Figure 6 and Supplementary Figure 2). By observing the primary root shootwards, fluorescence signals were limited to trichoblast cell files, which are always separated by at least one atrichoblast cell file (Supplementary Figure 4B). Interestingly, we found strong *CTPS2* expression again in the root tip cells of lateral roots, emphasizing the observed strong spatiotemporal expression (Figure 6 and Supplementary Figure 2).

### Auxin Treatment Increases CTPS2::GFP Fluorescence

**FIGURE 7:**
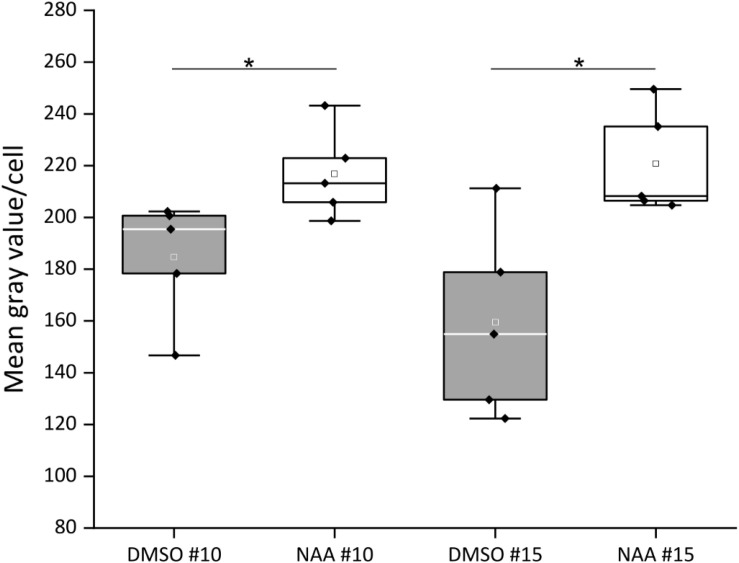
Quantification of CTPS2::GFP fluorescence intensity in trichoblasts after 1-naphthaleneacetic acid (NAA) application. Five-day-old seedlings, grown on 1/2 Murashige–Skoog (MS) agar plates, were transferred to 250 nM NAA or control [dimethyl sulfoxide (DMSO)] plates for 20 h. The fluorescence intensity of roots from five seedlings per treatment with10 cells per analyzed root is represented in box plots. White and gray rectangles represent mean value in DMSO and NAA-treated plants, respectively. Median is shown as white or black line in DMSO or NAA box plot, respectively. Asterisks indicate statistically different data (Student’s *t* test, *p* < 0.05).

Since the *CTPS2* promoter activity in columella cells revealed a similar fluorescence signal, compared to the established auxin reporter DR5::GFP, we aligned the nucleotide sequence of the auxin response element (TGTCTC) to the *CTPS2* promoter sequence used in this study (Ulmasov et al., 1995, 1997; Feng and Ma, 2017). Within the alignment, we found a repeat of the auxin response element (AuxRE) in the *CTPS2* promoter at position 652–665 upstream of exon 1 interrupted by the two bases CG (Supplementary Figure 3). This finding gives a hint that *CTPS2* may be auxin regulated. To support this finding, the two *CTPS2* reporter lines #10 and #15 were grown on 1/2 MS agar plates for 7 days and transferred to 1/2 MS agar plates supplemented with 250 nM 1-NAA or 250 nM DMSO. After 20 h growth, confocal laser scanning microscopy with subsequent fluorescence intensity analysis, using ImageJ (version 1.51j8), revealed indeed stronger GFP signal intensity in individual trichoblasts of NAA-treated plants (Supplementary Figure 4). Under control conditions, the mean gray value per cell of lines #10 and #15 were 184.7 and 159.4, respectively. NAA treatment increased the mean gray value per cell in both lines significantly, resulting in 216.8 for line #10 and 220.8 for line #15 (Figure 7). Notably, the increase in fluorescence intensity was most prominent in trichoblast cells in both lines; NAA treatment additionally induced fluorescence in cells of the root cortex and partially in atrichoblasts (Supplementary Figure 4). However, no significant upregulation of cellular fluorescence intensity was found for cells within the root cortex (data not shown).

## Discussion

During plant development, especially young tissues require enormous amounts of nucleotides for cell division and growth, e.g., for the synthesis of ribosomes. Therefore, it is assumed that *de novo* synthesis rates in seedlings and growing tissues are generally high (Zrenner et al., 2006). This hypothesis was supported by the finding of strong aspartate transcarbamoylase expression, the enzyme that commits the rate-limiting step in pyrimidine *de novo* synthesis, in *Arabidopsis* (Bassett et al., 2003; Chen and Slocum, 2008). A decrease in nucleotide *de novo* synthesis, caused by lower UMP synthase expression, increases pyrimidine salvage activity in growing potato tubers (Geigenberger et al., 2005). In contrast, differentiated cells maintain their nucleotide pools via salvage and compensate catabolic processes by minor *de novo* synthesis activity (Zrenner et al., 2006). Consequently, the external-supplied nucleoside cytidine, which can be imported in plants by ENT3 (Traub et al., 2007) and can be phosphorylated to CMP by uridine/cytidine kinase (Ohler et al., 2019), should allow for a compensation of lost CTP synthase activity in *ctps2* seedlings. However, the application of 1 mM cytidine did not overcome the germination phenotype of *ctps2* seeds (Figure 1). Together with the finding that siliques from *+/ctps2* plants contain nearly 25% aborted seeds, which also cannot be rescued by irrigation with 10 mM cytidine-containing water, but by complementation of *+/ctps2* plants with CTPS2 under control of the endogenous promoter, we conclude that the crucial function of CTPS2 lays in the embryo development (Figure 2). Transport of cytidine to the embryo, corresponding salvage activity or import competence appears to be insufficient to rescue embryo development.

It is known from work on *D. melanogaster* that egg chamber development goes along with a high demand for nucleotides and an accumulation of CTP synthase to allow for an increased ribosomal RNA (rRNA) synthesis (Aughey et al., 2016). Surprisingly, CTPS2::GFP fluorescence was restricted to the tip of columella cells in embryos of the heart and later stages. During embryo development, CTPS2::GFP fluorescence was observed in other embryonic tissues after 7 DAF (Figures 3, 4). In contrast to that, we noticed a relatively strong fluorescence in the peripheral endosperm of ovules 1.5 DAF (Figure 3, upper panel). Together with the finding of collapsed and embryo-free seeds in 8–10 DAF siliques of *+/ctps2-1* plants, it is likely that a knockout of *CTPS2* causes embryonic growth arrest at early embryo development. Andriotis and Smith (2019) found that impaired purine synthesis causes growth arrest and embryo abortion at the globular stage. This was also found for genes in the oxidative pentose phosphate pathway, providing precursors for phosphoribosyl-pyrophosphate (a cosubstrate in nucleotide *de novo* synthesis) synthesis and UMP synthase. Thus, we hypothesize that *CTPS2* is an *EMB* gene, in line with the results from Meinke (2020), causing growth arrest at early embryo development. Moreover, the knockout of enzymes in the pyrimidine *de novo* synthesis, which is encoded by a single gene, is embryo lethal (Schröder et al., 2005; Chen and Slocum, 2008). Since *Arabidopsis* possesses five CTP synthase genes, a spatiotemporal regulation of these is likely and would explain why *CTPS2* knockouts cannot be complemented by other CTP synthase isoforms. Certainly, one might argue that strong promoters as the UBQ promoter used in our approach, leading to relatively high expression of transgenes, should allow to complement missing expression. However, it might well be that the expression profile or the auxin responsiveness is required in this case. We also cannot rule out that a more intense search for complementing lines would be successful. Nevertheless, no other *Arabidopsis* CTP synthase harbors a repeat of the AuxRE in its promoter, except *CTPS2*. However, why is *CTPS2* expression restricted to the columella tip cells in the developing embryo? Stadler et al. (2005) established a mobile and membrane-bound GFP approach under control of the *At*SUC3 promoter, which allowed to monitor cell-to-cell movement. *At*SUC3 promoter activity was described in several sink tissues as well as the suspensor (Meyer et al., 2004). Nevertheless, Stadler et al. (2005) observed GFP fluorescence signals in embryos at the globular stage, concluding that the suspensor and early embryo are building a symplastic system. After the disconnection of suspensor and embryo (Bozhkov et al., 2005) and progression in embryo development, GFP fluorescence becomes more and more restricted to individual cell types, e.g., epidermis cells (Stadler et al., 2005). This implies that symplastic movement of macromolecules is no longer facilitated in the older embryo, but a tissue-specific connectivity by plasmodesmata must still remain (Stadler et al., 2005). It is accepted that molecules of a mass of up to 800 Da are able to diffuse freely via symplastic transport (Lalonde et al., 2004; Karmann et al., 2018). Since CTP has a mass of 483 Da, it is conceivable that CTPS2 produces CTP in embryonic columella cells, which is then symplastically distributed between all embryonic cells. The reduction in plasmodesmata during further development triggers CTPS2 activity in other cell types, resulting in GFP fluorescence in epidermis cells and cotyledons (Figure 4B).

The *CTPS2* expression pattern was similar to that of the auxin reporter DR5::GFP (Feng and Ma, 2017) during embryogenesis, and an AuxRE repeat was located in the *CTPS2* promoter region (Supplementary Figure 4). Maternal tissues supply the embryo with auxin until the globular stage, when the embryo is able to produce auxin by itself (Robert et al., 2018). With the fertilization of the ovum, suspensor cells express PIN7 for a directed auxin transport toward the embryo, and the auxin concentration is high at the embryos basal end (Friml et al., 2003). When the embryo starts independent auxin production in its apical cells, PIN1 expression is activated, resulting in an apical–basal auxin gradient, accumulating auxin in the basal end of the embryo and in upper suspensor cells (Friml et al., 2003; Vieten et al., 2005; Robert et al., 2013, 2018). Growth is a high demand time for nucleotides; thus, coupling of CTPS expression to the growth hormone auxin apparently makes sense. Symplastic coupling of embryonic cells could then allow for a distribution of nucleotides (CTP) within the embryo. At the same time, such system would possibly allow for better regulation compared to a salvage of phloem-derived cytidine, which according to our results does not take place.

In the root, DR5::GFP is expressed in the primary and lateral root tip, especially in columella cells, similar to the observations made in this study [Figures 5, 6 (Wabnik et al., 2011; Raya-González et al., 2018]. DR5::GFP and DR5::GUS are auxin-responsive promoter–reporter systems using repeats of a 11-bp sequence, containing the TGTCTC sequence in the synthetic promoter DR5 (Ulmasov et al., 1997; Ottenschläger et al., 2003). Therefore, it is conclusive that GFP fluorescence signals observed under control of the *CTPS2* promoter are not as intensive as DR5::GFP signals but can be intensified by auxin application (Figure 7 and Supplementary Figure 4). However, the columella is a highly dynamic tissue, which probably demands an extensive amount of CTP. Beneath its use as building block for RNA and DNA, in yeast, CTP is an essential precursor in membrane-phospholipid biosynthesis. Although CTP inhibits CTPS enzyme activity, in yeast, it was shown that the phosphorylation of CTPS stimulates its activity and increases endogenous CTP concentrations (Chang and Carman, 2008). Since the post-transcriptional regulation mechanisms and biochemical properties of plant CTPS are not completely understood yet (Daumann et al., 2018), it is possible that CTPS from *Arabidopsis* could be regulated similarly to yeast CTPS.

Already in mature embryos, a *CTPS2* expression pattern lining longitudinal rows of cells resembling trichoblast/atrichoblast pattern in roots of developing plants was observed (Figure 4B). Moreover, root hair formation is initiated by auxin and, in fact, we observed CTPS2 expression in root hair cells. The phytohormone stimulates AuxRE and thereby induces trichoblast-specific kinases, which initiate trichoblast elongation (Vissenberg et al., 2020). The major constituent of plant membranes is phosphatidylcholine, and its *de novo* synthesis requires CDP choline, delivered from the Kennedy pathway. One of the initial steps is the transfer of the cytidyl moiety from CTP to phosphocholine (Kennedy, 1958; Caldo et al., 2019). The transition from a trichoblast cell into a root hair, which is in the end still one cell, requires enormous amounts of membrane phospholipids and thus CTP (Kinney, 1993). Together with our finding of high *CTPS2* promoter activity in trichoblasts (Figure 6 and Supplementary Figure 2), *CTPS2* transcript was found to be upregulated in an RNA-seq analysis on *Arabidopsis* root hair cells (Huang et al., 2017).

Taken together, we conclude from our data that *CTPS2* is required for embryo development. We propose that auxin may act on the transcriptional regulation of *CTPS2* in embryo and seedling roots and thus affects their development.

## Data Availability Statement

The original contributions presented in the study are included in the article/Supplementary Material, further inquiries can be directed to the corresponding author.

## Author Contributions

DH generated complementation and promoter–reporter gene lines and analyzed seed development and germination. DH and DS performed confocal microscopy. DS advised auxin treatment experiments and quantification of fluorescence intensity, conducted by DH. All authors contributed to the research design and wrote the manuscript.

## Conflict of Interest

The authors declare that the research was conducted in the absence of any commercial or financial relationships that could be construed as a potential conflict of interest.
