# Supplementary material for: CTP Synthase 2 From Arabidopsis thaliana Is Required for Complete Embryo Development
Source: Front Plant Sci. 2021 Apr 15;12:652434. doi: 10.3389/fpls.2021.652434 (PMC8082242; doi:10.3389/fpls.2021.652434)
Supplement: Supplementary file 1 [file Data_Sheet_1.pdf]

Supplemental Data

Hickl et al.

“CTP-Synthase 2 from *Arabidopsis thaliana* is required for complete embryo development”

Supplemental Tables

Table S1: Primers used in this study

Designation	s-sequence (5’-3’)	as-sequence (5’-3’)	Description
CTPS2_Promoter	attgttcatcaccactcttctctc	aatcgtttttgtctctgcttcttc	gDNA amplification
CTPS2_Full-length	aatttgaaccttgggctggttaca	gtggtgaagcccgtttccattg	gDNA amplification
CTPS2_Promoter	ggggacaagtttgtacaaaaaagcaggcttaattgttcatcaccactctt	ggggaccactttgtacaagaaagctgggtaaatcgtttttgtctctgctt	att-site attachment
CTPS2_Full-length	ggggacaagtttgtacaaaaaagcaggcttaaatttgaaccttgggctgg	ggggaccactttgtacaagaaagctgggtagtggtgaagcccgtttccat	att-site attachment
+/ctps2-1	gtaagtcttccttgaccctaagcg		Left border
+/ctps2-1	aaaagctcatgaagcgatcctaag		Right border
+/ctps2-2	gctcatatctccgtcgttacaaac		Left border
+/ctps2-2	ccaggtatttgtactacctcctca		Right border
GK_o8474	ataataacgctgcggacatctacatttt		LB-primer
CTPS2_700	atgatatcttgttgtaatcctagt		Sequencing
CTPS2_1400	atgcaaacttccacaacacaaaca		Sequencing
CTPS2_2100	gtagctatgagatcctttttcaca		Sequencing
CTPS2_2800	acaggtgatattgaatctatg		Sequencing
CTPS2_3500	gttcctctgcttttaagggt		Sequencing
CTPS2_4200	catagagtgggttgcagcta		Sequencing
CTPS2_4900	aaatctatataacttctaatctct		Sequencing
CTPS2_5600	ctcgagaaaggaacaattttactg		Sequencing
Ef1α	gagaccaccaagtactactgcac	gttggtcccttgtaccagtcaag	Quality control

Supplemental Figures

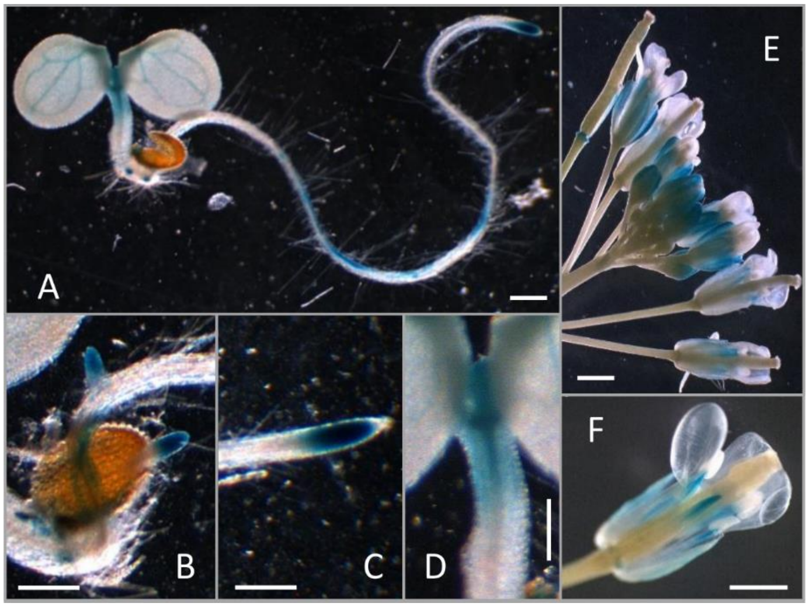

Figure S1. Histochemical staining of CTPS2::GUS lines in seedlings and in reproductive tissues. (A-D) Five day old seedlings grown on ½ MS agar plates. (A) Seedling with GUS staining in the primary root, shoot apical meristem and vasculature tissue. (B, C) Root tips of primary (C) and secondary roots (B) show marked GUS staining. (D) CTPS2::GUS signals in the shoot apical meristem. (E, F) During reproductive growth phase CTPS2::GUS signals were observed in filaments of the flower. Scale bar = 1 mm

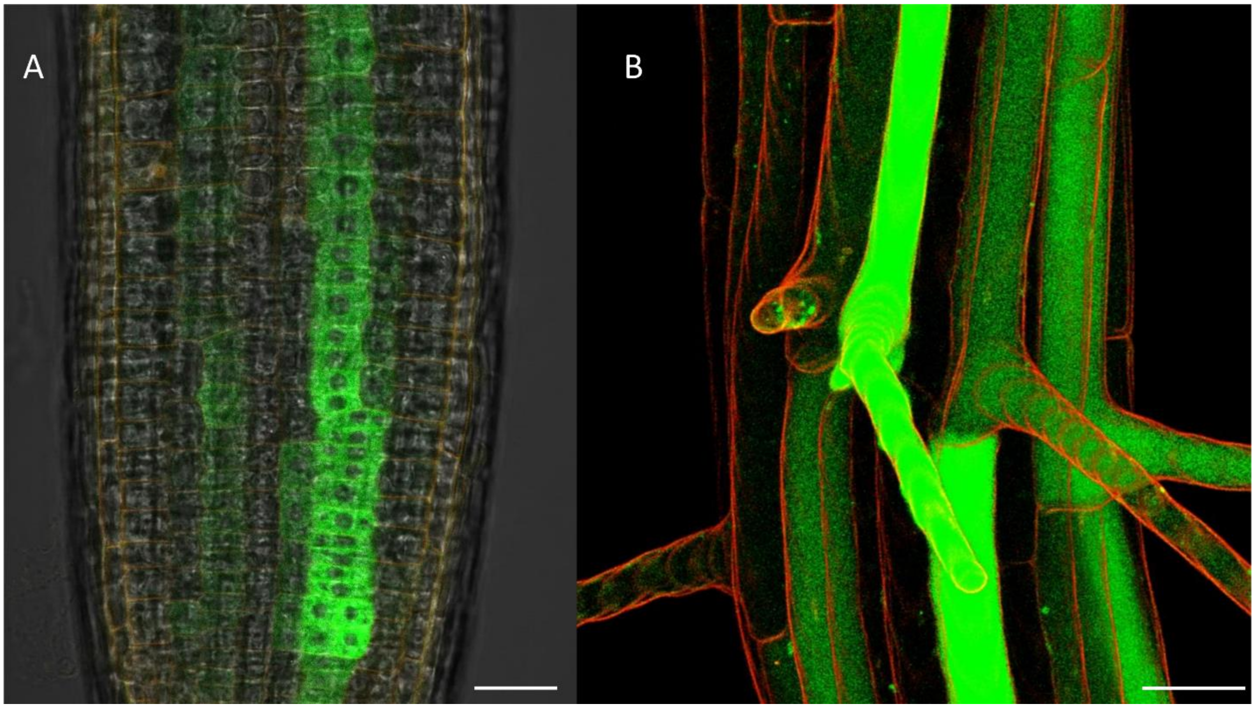

Figure S2. CTPS2 promoter activity is strong in trichoblasts and root hair cells. Plants were grown on ½ MS agar plates for five days, transferred to slides and cell walls were stained with propidium iodide. Confocal laser scanning microscopy was conducted with a Zeiss LSM880 AxioObserver SP7. Scale bar 25 µm. (B) 39 pictures were used as maximum projection with 4 µm frame for each picture. (A) primary root with stained trichoblasts before root hair outgrowth. (B) Stained root hairs

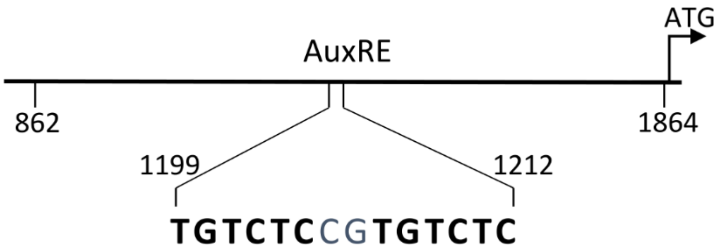

Figure S3. Auxin response element found in the promoter region of *CTPS2*. The sequence was included in all promoter activity studies conducted in this work.

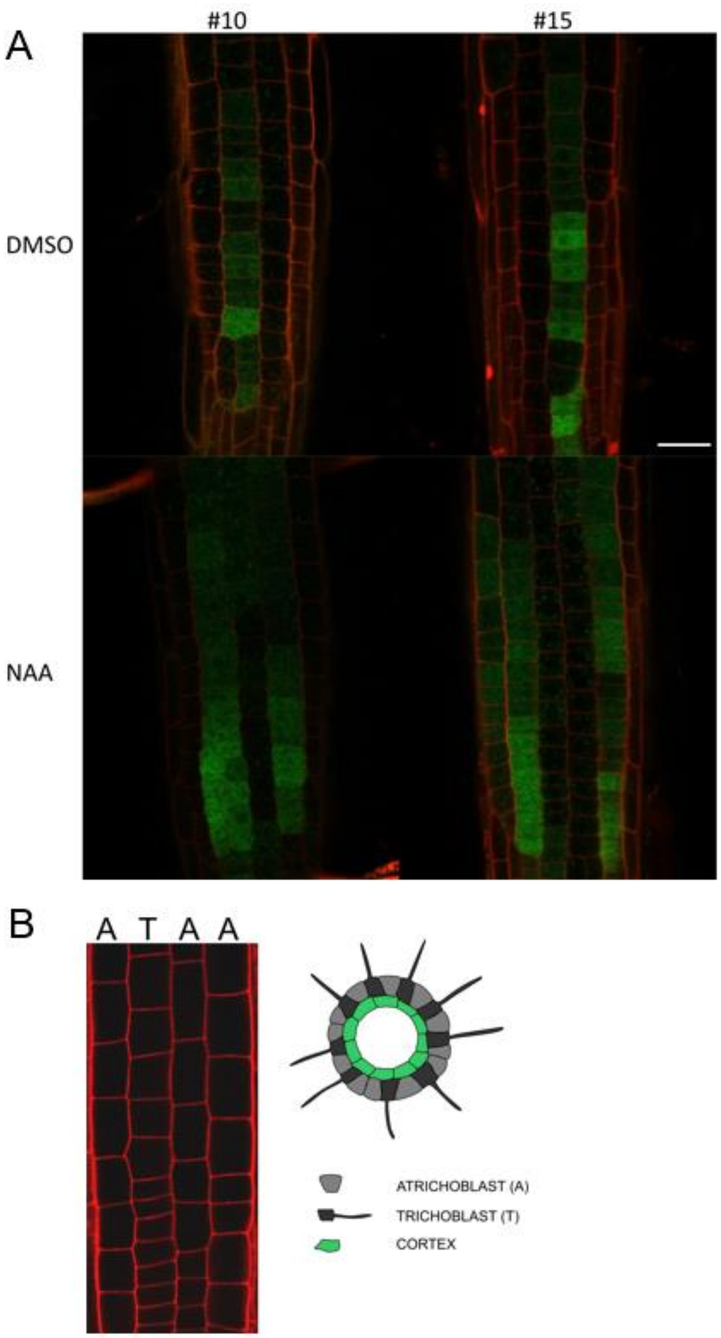

Figure S4. NAA treatment increases CTPS2::GFP fluorescence. Seedlings of the two independent CTPS2::GFP lines #10 and #15 were grown on ½ MS agar plates for 5 days and transferred to plates containing DMSO as control or 250 nM NAA for 20 h. (A) DMSO (mock) treated roots show typical GFP fluorescence in trichoblasts. NAA treatment increased the GFP signal in trichoblast cells. GFP staining is also visible cortex cells and some atrichoblasts. (B) Scheme of the organization of rhizodermis and root cortex cells. Propidium iodide was used to stain cell walls. Scale bar = 25 µm.
